# Melatonergic Signaling Sustains Food Allergy Through FcεRI Recycling

**DOI:** 10.34133/research.0418

**Published:** 2024-07-22

**Authors:** Youxia Wang, Xinmei Zhang, Ifen Hung, Chunxue Liu, Wenkai Ren, Liangpeng Ge, Hao Wang

**Affiliations:** ^1^ State Key Laboratory of Livestock and Poultry Breeding, Guangdong Laboratory of Lingnan Modern Agriculture, College of Animal Science, South China Agricultural University, Guangzhou, China.; ^2^ Anyou Biotechnology Group Co. Ltd., Taicang, China.; ^3^ Joint Laboratory of Functional Nutrition and Animal Health, Centree Bio-tech (Wuhan) Co. Ltd., Wuhan, China.; ^4^ National Center of Technology Innovation for Pigs; Chongqing Academy of Animal Sciences; Key Laboratory of Pig Industry Science, Ministry of Agriculture, Chongqing, China.; ^5^ College of Animal Science and Veterinary Medicine, Henan Institute of Science and Technology, Xinxiang, Henan, China.

## Abstract

The prevalence of food allergies is increasing dramatically and causing serious public health concerns. Notably, melatonin metabolism imbalance in patients with food allergies; however, the role of melatonin in food allergies remains unclear. Here, we demonstrated that melatonin suppresses food allergy responses and reprograms the gut microbiota of food-allergic mice, while melatonin aggravates food allergy during gut microbiota depletion. Mechanistically, melatonin boosts the degranulation of mast cells by up-regulating the expression of membrane high-affinity immunoglobulin E (IgE) receptor (FcεRI). Melatonin increases the mRNA expression of Rabenosyn-5 (a component of factors for endosome recycling and Rab interactions) through melatonin receptor 2 (MT2)–extracellular signal-regulated kinase (ERK) signaling, thereby driving the recycling of FcεRI and elevating the abundance of membrane FcεRI. Likewise, the inhibition of MT2 attenuates melatonin-induced food allergy in mice with gut microbiota depletion. Collectively, our finding provides insights into the pathogenesis of food allergies and provides a potential therapeutic target for the prevention and treatment of food allergies.

## Introduction

Food allergies are pathologic immune responses triggered by food antigens [1]. The prevalence of food allergies has increased dramatically over the past 2 decades, affecting more than 500 million people worldwide. [2]. Food allergies cause multiple clinical symptoms, including diarrhea, eczema, and asthma, as well as shock and other life-threatening situations, in severe cases [3]. Multiple factors are involved in the pathological mechanism of food allergies, including immune cells [e.g., dendritic cells (DCs), T cells, and mast cells] [4], epithelial barrier [5], and gut microbiota [6]. Notably, the pathology of food allergies reveals unique metabolic characteristics, including altered tryptophan metabolism [7]. Melatonin (a tryptophan-associated metabolite) metabolism is disrupted in patients with food allergies [8–10]. Although accumulating evidence supports that cellular metabolism shapes the function of the immune system [11–15], the role of metabolic alterations (e.g., melatonin) in the pathogenesis of food allergies remains to be elucidated.

Melatonin has been considered as a rhythm regulator [16] and has multiple physiological functions in circadian rhythms [17], immune system [18], and gut microbiota [19]. Despite that melatonin treatment could alleviate symptoms of allergic asthma [20], the symptoms of a variety of allergic diseases (e.g., allergic rhinitis [21] and bronchial asthma [22]) are intense during the peak of melatonin secretion. Thus, the effects of melatonin on food allergies and the underlying mechanism for melatonin to affect food allergies are still obscure. In addition, multiple immune cells (e.g., T cells, macrophages, and mast cells) possess melatonergic systems [2]. In particular, mast cells (key effector cells of food allergies) synthesize and release melatonin, and the expression of melatonin receptors is increased during activation. Furthermore, our previous studies showed that melatonin shapes the fate of immune cells [18]. However, the potential role of melatonergic signaling in the activation of mast cells and the pathogenesis of food allergies remain unclear.

Here, we demonstrated that melatonin treatment alleviates allergy symptoms and reprograms the gut microbiota of food-allergic mice, while melatonin aggravates allergy symptoms in mice with microbiota depletion. Mechanistically, melatonin promotes high-affinity immunoglobulin E (IgE) receptor (FcεRI) recycling by activating the melatonin receptor 2 (MT2)/extracellular signal-regulated kinase (ERK)/Rabenosyn-5 pathway, resulting in degranulation of mast cells. Our findings provide evidence that melatonergic signaling is a potential target for preventing and treating food allergies.

## Results

### Melatonin suppresses food allergies induced by OVA in mice

**Fig. 1.**
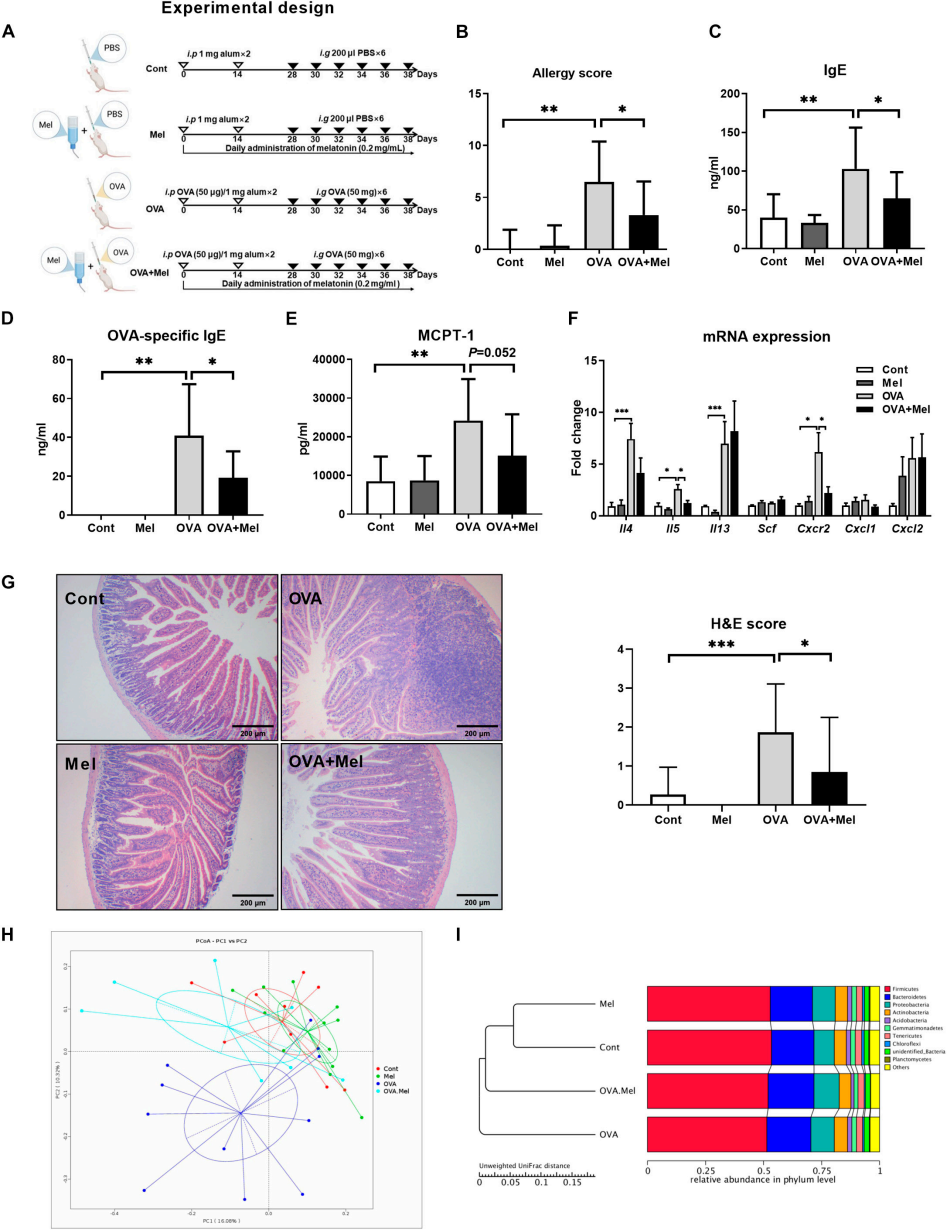
Melatonin alleviates OVA-induced food allergies. (A) Experimental protocol. OVA and OVA + Mel mice were sensitized twice within a 2-week interval by intraperitoneal (i.p) injection of 50 μg of OVA in Al(OH)_3_ (alum) followed by intragastric (i.g) gavage with 50 mg of OVA in PBS 6 times within 12 days. Cont and Mel mice were injected with PBS/alum and gavaged by PBS. Mel and OVA + Mel mice were treated with 0.2 mg/ml melatonin in drinking water. (B) Clinical allergy score of mice (*n* = 13 to 15). (C to E) Serum concentration of IgE, OVA-specific IgE, and MCPT-1 in mice (*n* = 5 to 13). (F) Relative mRNA expression of T_H_2 cytokines, Scf, Cxcr2, Cxcl1, and Cxcl2 in the jejunum of mice (*n* = 4 to 12). (G) The jejunum tissue was stained with H&E (*n* = 13 to 15). (H) PCoA based on the operational taxonomic unit (OTU)-level microbiota abundance data of the first 2 components (*n* = 9 to 11). (I) Unweighted UniFrac distance based on the relative abundance in phylum level of the mice gut microbiota (*n* = 9 to 11). Data were analyzed with one-way ANOVA (E) or Kruskal–Wallis (B to D, F, and G). (B) to (E) and (G) are represented as mean ± SD. (F) is represented as mean ± SEM. *P < 0.05, **P < 0.01, ***P < 0.001.

Multiple evidences support that melatonin metabolism is altered in patients with allergic diseases [7–9], and intraperitoneal injections or airway irrigation of melatonin relieves lung inflammation of allergic diseases [23]; however, the role of oral melatonin in food allergies remains obscure. To uncover this, 0.2 mg/ml melatonin was supplemented into the drinking water of mice with food allergies (Fig. 1A). Notably, melatonin treatment eased ovalbumin (OVA)-induced food allergy symptoms (Fig. S1A to D) and reduced the allergy score (Fig. 1B). After reexposure to food antigens, antigen-presenting cells induce a T helper 2 (T_H_2) response that drives IgE switching in B cells and promotes the degranulation of mast cells [24]. Then, mast cells release inflammatory mediators [e.g., mast cell protease 1 (MCPT-1) and histamine] rapidly and trigger clinical symptoms [24]. Moreover, melatonin reduced the level of total and antigen-specific IgE responses and MCPT-1 in serum (Fig. 1C to E).

Sensitization of food allergies is a type of T_H_2 immune response mounted against food antigens [24]. Importantly, melatonin treatment significantly reduced the expression of jejunal *Il 5* in OVA-challenged mice (Fig. 1F). Migration of mast cells to peripheral tissues is crucial for them to perform effector functions [25]. The mRNA expression of Cxcr2 (a chemokine receptor of mast cell) in the jejunum in mice with food allergy was also inhibited by melatonin (Fig. 1F). Compared with the OVA group, the jejunal inflammation was significantly alleviated in the OVA + Mel group (Fig. 1G).

Growing evidence points to an important role of gut microbiota in food allergies [26], and melatonin regulates various pathophysiological events through remodeling gut microbiota [19,27–29]. Thus, 16*S* rDNA amplicon sequencing was used to determine fecal microbiota compositions. Principal coordinate analysis (PCoA) and unweighted UniFrac distance metric matrices showed a clear separation of the gut microbiota between the Cont and OVA groups, while the OVA + Mel group has a similar structure to that of the Cont and Mel groups (Fig. 1H and I). Furthermore, melatonin modulated the α-diversity and composition of gut microbiota at phylum, order, and genus level (Fig. S2A to C). Collectively, melatonin supplementation suppresses food allergy responses and markedly reprograms the gut microbiota of food-allergic mice.

### Melatonin aggravates food allergies in mice with microbiota depletion

**Fig. 2.**
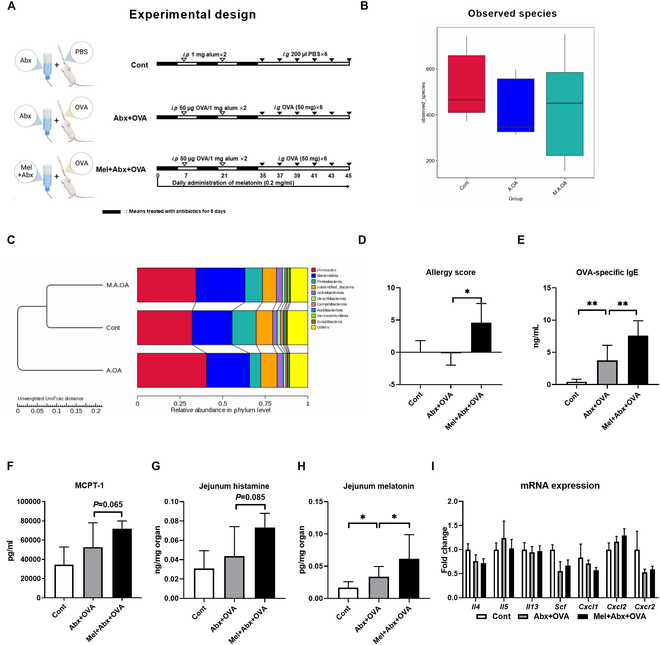
Melatonin aggravates food allergies in mice with gut microbiota depletion. (A) Experimental protocol. Abx + OVA and Mel + Abx + OVA mice were sensitized twice within a 2-week interval by intraperitoneal injection of 50 μg of OVA in Al(OH)_3_ (alum) followed by intragastric gavage with 50 mg of OVA in PBS 6 times within 12 days. Cont mice were injected with PBS/alum and gavaged by PBS. All mice were treated with antibiotics for 3 times (each time for 5 days). (B) Number of observed species in the mice gut microbiota (*n* = 10). (C) Unweighted UniFrac distance based on the relative abundance in phylum level of the mice gut microbiota (*n* = 10). (D) Clinical allergy score of mice (*n* = 15). (E and F) Serum concentration of OVA-specific IgE and MCPT-1 in mice (*n* = 8). (G and H) Concentration of histamine and melatonin in the jejunum of mice (*n* = 5 to 8). (I) Relative mRNA expression of T_H_2 cytokines, Scf, Cxcr2, Cxcl1, and Cxcl2 in the jejunum of mice (*n* = 6 to 8). Data were analyzed with one-way ANOVA (D and I) or Kruskal–Wallis (E to H). (D) to (H) are represented as mean ± SD. (I) is represented as mean ± SEM. *P < 0.05, **P < 0.01.

We have shown that melatonin suppresses food anaphylactic reactions and remodels gut microbiota in the food allergy model. Therefore, we hypothesized that melatonin might exert its anti-allergy effect through gut microbiota. Antibiotic mixtures (Abx) were used to clear the gut microbiota (Fig. 2A). Although showing no effect on the number of observed species (Fig. 2B) and the α-diversity, melatonin affected the composition of gut microbiota in food-allergic mice with Abx treatment (Fig. 2C and Fig. S3A to D). Interestingly, despite the high level of sensitization, Abx + OVA mice did not develop growth retardation, diarrhea, or common symptoms of food allergy (Fig. 2D and Fig. S4A to D), indicating that specific gut microbes drive food allergies in mice. Unexpectedly, melatonin treatment aggravated food allergy symptoms (Fig. 2D and Fig. S4A to D) in mice with Abx treatment.

Therefore, we then explored how melatonin sustains food allergies during the Abx treatment. Melatonin elevated the concentration of serum OVA-specific IgE (Fig. 2E). Interleukin-4 (IL-4) is essential for IgE switching. Moreover, switching the B cell class to IgE results in the production of ε-germline transcripts [30,31]. Our results showed that melatonin up-regulated the mRNA expression of IL-4 receptor (IL-4R) and ε-germline, indicating that melatonin promotes the production of IgE (Fig. S4E). Furthermore, the level of serum MCPT-1 and histamine in the jejunum also increased by melatonin treatment (Fig. 2F and G). These results suggested that melatonin promoted degranulation of mast cells in mice with Abx treatment. Consistently, the concentration of melatonin in the jejunum of mice with Abx treatment also increased with melatonin treatment (Fig. 2H). However, melatonin did not influence the jejunal mRNA expression of T_H_2 cytokines (IL-4, IL-5, and IL-13) (Fig. 2I) in mice with Abx treatment. Also, there was no change in the mRNA expression of mast cell growth factor (SCF), chemokines (Cxcl1 and Cxcl2), and chemokine receptor (Cxcr2) (Fig. 2I) for mast cell differentiation and migration. These observations collectively supported the notion that melatonin is involved in the pathogenesis of food allergies in mice with gut microbiota dysbiosis.

### Melatonin promotes the degranulation of mast cells by up-regulating the expression of FcεRI

**Fig. 3.**
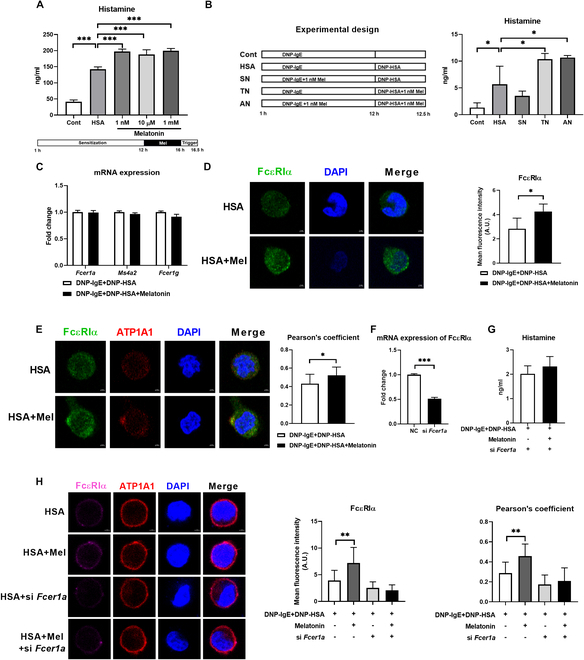
Melatonin promotes mast cell degranulation through FcεRI. (A and B) Secretion of histamine from mast cells treated with or without melatonin (*n* = 3 to 4). (C) mRNA expression of FcεRIα, MS4A2, and FcεRIγ of mast cells treated with or without melatonin (*n* = 5). (D) Confocal microscopy of FcεRIα (green) in mast cells treated with or without melatonin (*n* = 4). (E) Confocal microscopy images show the colocalization of FcεRIα with ATP1A1 in mast cells treated with or without melatonin (*n* = 12 to 13). (F) Relative mRNA expression of FcεRIα in mast cells treated with or without si *Fcer1a*. (G) Secretion of histamine from mast cells treated with or without si *Fcer1a* in the presence of melatonin (1 nM) (*n* = 4 to 6). (H) Confocal microscopy of FcεRIα (purple) and the colocalization of FcεRIα with ATP1A1 in mast cells treated with or without si *Fcer1a* in the presence of melatonin (1 nM) (*n* = 9 to 11). Data were analyzed with unpaired *t* tests (E and G), Mann–Whitney *U* test (C, D, and F), one-way ANOVA (H), or Kruskal–Wallis (A and B). (A), (B), (D), (E), (G), and (H) are represented as mean ± SD. (C) and (F) are represented as mean ± SEM. *P < 0.05, **P < 0.01, ***P < 0.001.

Mast cells are the main effector cells of food allergies [32], and studies have shown that the expression of melatonin receptors was increased in activated mast cells [33]. Therefore, we investigated the roles of melatonin on mast cell degranulation in vitro. The rat basophilic leukemia cell MC lines, RBL-2H3, have been widely used as a model for mast cell IgE-mediated degranulation [34,35]. Activated RBL-2H3 cells showed altered melatonin metabolism, including the increase of 5-hydroxytryptophan (5-HTP), 5-hydroxytryptamine (5-HT), and melatonin (Fig. S5A), as well as the up-regulation of acrylamide-O-methyltransferase (ASMT) activity (Fig. S5B and C), suggesting that melatonin is involved in the activation of mast cells. Interestingly, melatonin pretreatment (1 nM, 10 μM, or 1 mM) promoted the secretion of histamine (Fig. 3A) from activated mast cells. Two processes are involved in the activation of mast cells, including sensitization to monoclonal anti-dinitrophenyl IgE (anti-DNP-IgE) and stimulation with DNP-albumin human (DNP-HSA). To further uncover whether melatonin acts during sensitization or stimulation, mast cells were treated with melatonin (1 nM) during sensitization and stimulation, respectively, or treated with melatonin during both sensitization and stimulation (Fig. 3B). Notably, the secretion of histamine is enhanced by supplementation of melatonin during stimulation of mast cells (Fig. 3B), indicating that melatonin mainly affects mast cell degranulation during stimulation.

The degranulation of mast cells is triggered by the binding of antigen-specific IgE to FcεRI [32], and the expression of FcεRI is crucial for the degranulation of mast cells [36]. We thus assessed the expression of FcεRI in mast cells after melatonin treatment. Melatonin did not influence the mRNA expression of FcεRI (Fig. 3C) but increased the protein abundance of total FcεRIα and membrane FcεRIα (Fig. 3D and E) in activated mast cells. To investigate whether the action of melatonin on mast cell degranulation depends on FcεRIα, small interference RNA was used to knock down the mRNA expression of FcεRIα in mast cells (Fig. 3F). As expected, FcεRIα silencing blocked the effect of melatonin on histamine secretion and total and membrane FcεRIα abundance in activated mast cells (Fig. 3G and H). Collectively, these results indicated that melatonin promotes the degranulation of mast cells largely dependent on the expression of membrane FcεRI.

### Melatonin promotes FcεRI recycling to enhance its stability

**Fig. 4.**
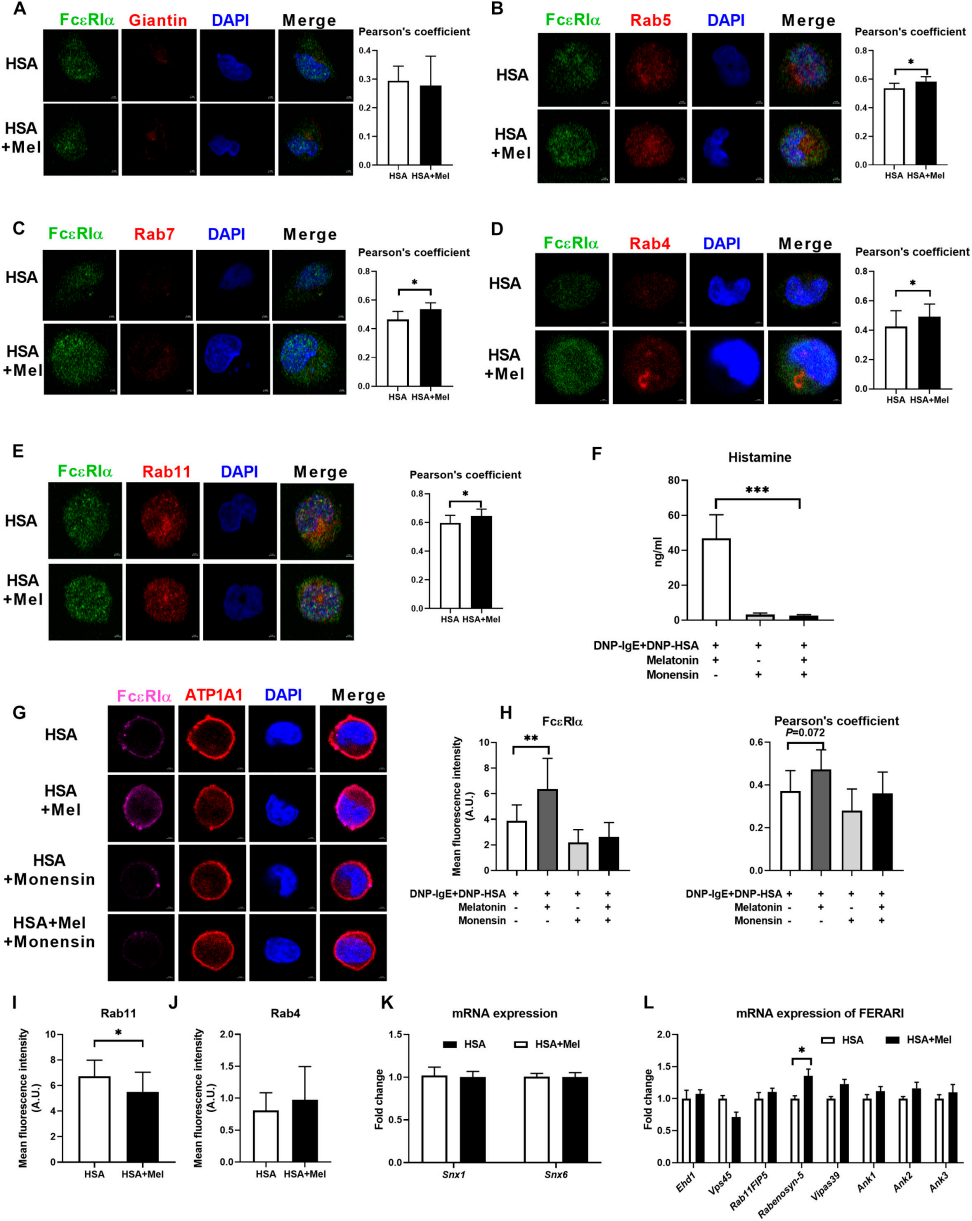
Melatonin up-regulates the abundance of membrane FcεRI through recycling endosomes. (A to E) Confocal microscopy images show the colocalization of FcεRIα with giantin, Rab5, Rab7, Rab4, and Rab11 in mast cells treated with or without melatonin (*n* = 7 to 15). (F) Secretion of histamine from mast cells treated with or without recycling inhibitor (monensin) in the presence of melatonin (*n* = 6). (G and H) Confocal microscopy of FcεRIα (purple) and the colocalization of FcεRIα with ATP1A1 in mast cells treated with or without recycling inhibitor (monensin) in the presence of melatonin (*n* = 10 to 12). (I and J) Confocal microscopy of Rab11 and Rab4 in mast cells treated with or without melatonin (*n* = 24 to 25). (K and L) Relative mRNA expression of SNX1, SNX6, and FERARI in mast cells treated with or without melatonin (*n* = 4). Data were analyzed with unpaired *t* tests (A to E and I to L) or one-way ANOVA (F and H). (A) to (J) are represented as mean ± SD. (K) and (L) are represented as mean ± SEM. *P < 0.05, **P < 0.01, ***P < 0.001.

The abundance of cell surface proteins relies on the secretory pathway and the endolysosomal network [37,38], and about 70 to 80% of endocytosed material is recycled back to the plasma membrane [39]. The endocytosed material is sorted and transported into different intracellular trafficking pathways, including transportation to the late endosomes or lysosomes for degradation, to the trans-Golgi network, or to recycling endosomes for recycling back to the plasma membrane [40]. Thus, we assessed the colocalization of FcεRIα with Golgi apparatus and endolysosomal markers, including giantin (Golgi marker), Rab5 (early endosome marker), Rab7 (late endosome marker), Rab4 (fast cycling endosome marker), and Rab11 (slow cycling endosome marker) (Fig. 4A to E). The colocalization of FcεRIα with Rab5, Rab7, Rab4, and Rab11 was elevated by melatonin in activated mast cells (Fig. 4B to E). Thus, we suspected that melatonin elevated membrane FcεRIα expression by enhancing FcεRIα recycling. Consistently, the effects of melatonin on histamine secretion and membrane FcεRI abundance were blocked by monensin (a recycling inhibitor) in activated mast cells (Fig. 4F to H).

Membrane protein from sorting endosomes can be recycled back to the plasma membrane by a fast recycling pathway (mediated by Rab-4) and a slow process (mediated by Rab-11) via late recycling endosomes [41]. However, melatonin-treated cells showed no change in Rab4 and even lower Rab11 (Fig. 4D, E, I, and J). Factors for endosome recycling and Rab interactions (FERARI) and sorting nexin (SNX1) negatively regulate the size of Rab11-positive structures [39]. Moreover, FERARI and SNX6 coordinate cargo flow through sorting endosomes in recycling [42]. To further test whether SNX1, SNX6, and FERARI are involved in Mel/Rab11-dependent FcεRI recycling, we determined the mRNA expression of SNX1, SNX6, and FERARI members (Fig. 4K and L). Of note, melatonin treatment significantly increased the mRNA expression of Rabenosyn-5, a component of FERARI (Fig. 4L). In summary, melatonin promotes the degranulation of mast cells by enhancing the recycling of FcεRIα, which may be mediated by Rabenosyn-5.

### Melatonin promotes FcεRI recycling through the MT2/ERK pathway

**Fig. 5.**
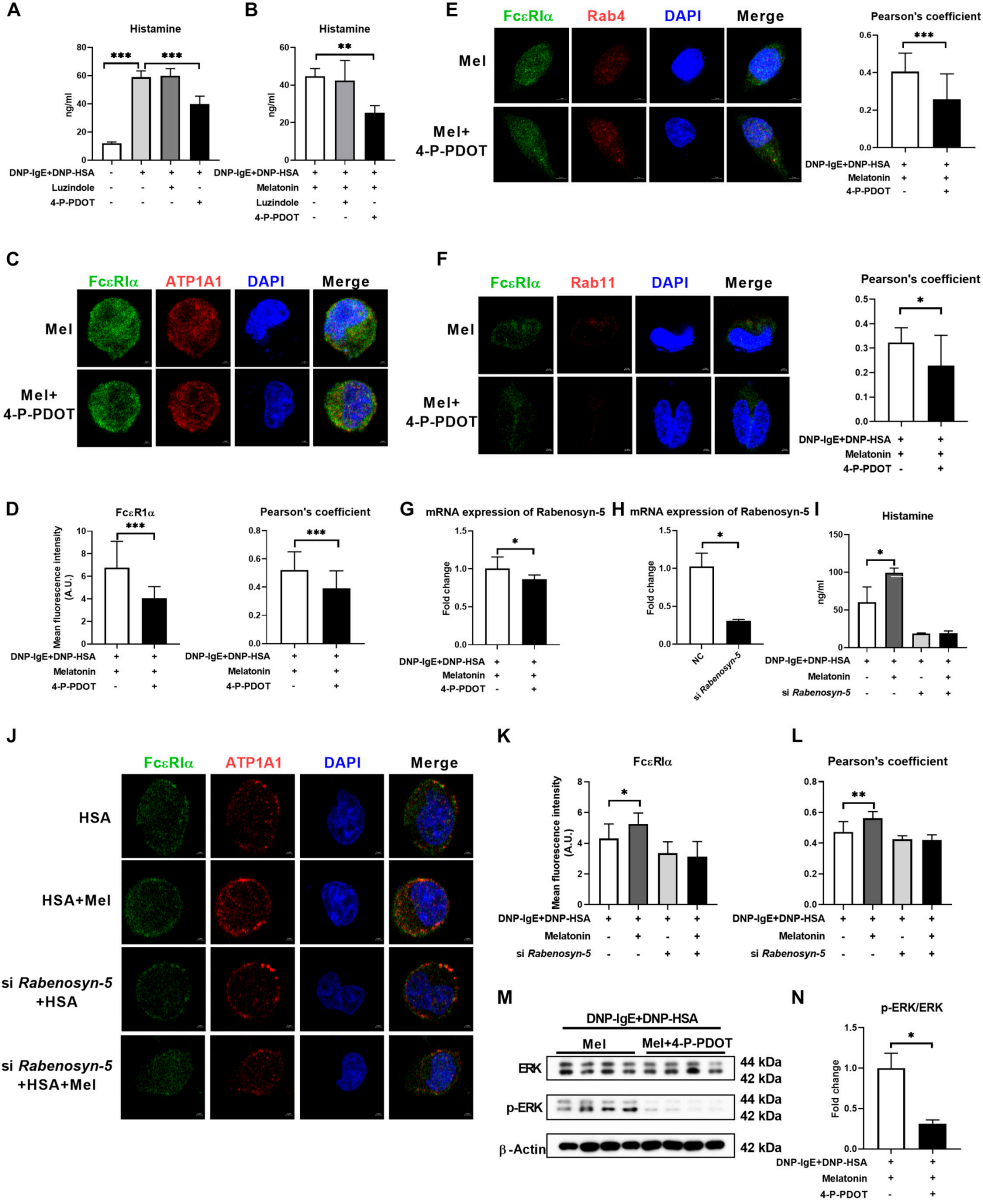
Melatonin promotes the recycling of FcεRI through the MT2/ERK/Rabenosyn-5 axis. (A) Secretion of histamine from mast cells treated with or without MT1 inhibitor (luzindole) and MT2 inhibitor (4-P-PDOT) (*n* = 3 to 4). (B) Secretion of histamine from mast cells treated with or without MT1 inhibitor (luzindole) and MT2 inhibitor (4-P-PDOT) in the presence of melatonin (*n* = 4). (C and D) Confocal microscopy of FcεRIα (green) and the colocalization of FcεRIα with ATP1A1 in mast cells treated with or without MT2 inhibitor (4-P-PDOT) in the presence of melatonin (*n* = 18). (E and F) Confocal microscopy images show the colocalization of FcεRIα with Rab4 and Rab11 in mast cells treated with or without MT2 inhibitor (4-P-PDOT) in the presence of melatonin (*n* = 15 to 27). (G) Relative mRNA expression of Rabenosyn-5 in mast cells treated with or without MT2 inhibitor (4-P-PDOT) in the presence of melatonin (*n* = 8). (H) Relative mRNA expression of Rabenosyn-5 in mast cells treated with or without si *Rabenosyn-5* (*n* = 3). (I) Secretion of histamine from mast cells treated with or without si *Rabenosyn-5* in the presence of melatonin (*n* = 3 to 6). (J to L) Confocal microscopy of FcεRIα (green) and the colocalization of FcεRIα with ATP1A1 in mast cells treated with or without MT2 inhibitor (4-P-PDOT) in the presence of melatonin (*n* = 8). (M and N) Protein abundance of ERK and p-ERK in mast cells treated with or without MT2 inhibitor (4-P-PDOT) in the presence of melatonin (*n* = 4). Data were analyzed with unpaired *t* tests (D to G), Mann–Whitney *U* test (H and N), one-way ANOVA (K and L), or Kruskal–Wallis (A and B). (A) to (F) and (I) to (N) are represented as mean ± SD. (G) and (H) are represented as mean ± SEM. *P < 0.05, **P < 0.01, ***P < 0.001.

We then explored the mechanism by which melatonin influences FcεRI recycling. We used luzindole and 4-phenyl-2-propionamidotetralin (4-P-PDOT) to inhibit MT1 and MT2 in activated mast cells, respectively (Fig. 5A and B). Notably, 4-P-PDOT inhibited the secretion of histamine in the control group and melatonin treatment group (Fig. 5A and B), indicating that melatonin promotes the degranulation of mast cells through MT2. We then assessed the expression and recycling of FcεRIα after MT2 inhibitor (4-P-PDOT) treatment and found that 4-P-PDOT inhibited membrane FcεRIα abundance (Fig. 5C and D) and the colocalization of FcεRIα with Rab4 and Rab11 (Fig. 5E and F) in activated mast cells. Notably, the mRNA expression of Rabenosyn-5 was also reduced by 4-P-PDOT in melatonin-treated mast cells (Fig. 5G). These results suggested that MT2–Rabenosyn-5 signaling mediates the promotion effects of melatonin on FcεRI recycling and mast cell degranulation.

To further validate the role of Rabenosyn-5 in regulating FcεRI recycling and mast cell degranulation, we transfected RBL-2H3 cells with si *Rabenosyn-5* to inhibit the expression of Rabenosyn-5 (Fig. 5H). Like recycling endosome inhibition and MT2 inhibition, *Rabenosyn-5* knockdown blocked the effect of melatonin on histamine secretion and membrane FcεRIα abundance in RBL-2H3 cells (Fig. 5I to L). These results demonstrated that melatonin promotes FcεRI recycling through the MT2/Rabenosyn-5 axis. Subsequently, we investigated how melatonin–MT2 signaling influences FcεRI recycling. Melatonin leads to ERK activation via MT2 [2], and ERK mediates endocytic recycling [43]. Consistently, 4-P-PDOT treatment inhibited the phosphorylation of ERK (Fig. 5M and N). Collectively, melatonin promotes FcεRI recycling through the MT2/ERK/Rabenosyn-5 axis.

### MT2 inhibition attenuates food allergies in melatonin-treated mice with microbiota depletion

**Fig. 6.**
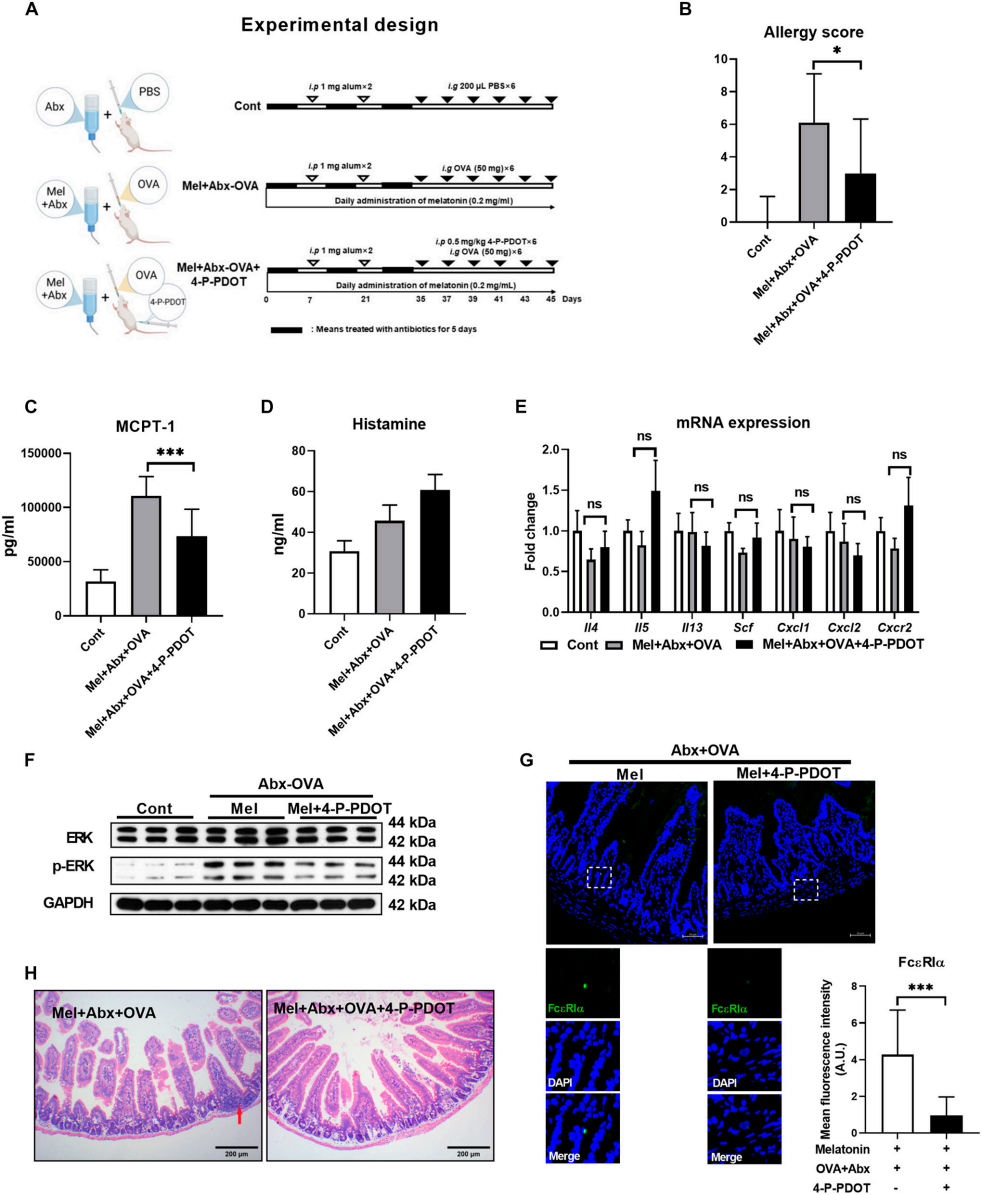
MT2 inhibition attenuates melatonin-induced food allergies in mice with gut microbiota depletion. (A) Experimental protocol. Mel + Abx + OVA and Mel + Abx + OVA + 4-P-PDOT mice were sensitized twice within a 2-week interval by intraperitoneal injection of 50 μg of OVA in Al(OH)_3_ (alum) followed by intragastric gavage with 50 mg of OVA in PBS 6 times within 12 days. Mel + Abx + OVA and Mel + Abx + OVA + 4-P-PDOT mice were treated with 0.2 mg/ml melatonin in drinking water. Mel + Abx + OVA + 4-P-PDOT mice were intraperitoneally injected with 0.5 mg/kg 4-P-PDOT 30 min before intragastric challenge with OVA. Cont mice were injected with PBS/alum and gavaged by PBS. All mice were treated with antibiotics for 3 times (each time for 5 days). (B) Clinical allergy score of mice (*n* = 10 to 12). (C and D) Serum concentration of MCPT-1 and histamine in mice (*n* = 8 to 9). (E) Relative mRNA expression of T_H_2 cytokines, Scf, Cxcr2, Cxcl1, and Cxcl2 in the jejunum of mice (*n* = 6 to 7). (F) Protein abundance of ERK and p-ERK in mice treated with or without MT2 inhibitor (4-P-PDOT) (*n* = 4). (G) Confocal microscopy of FcεRIα (green) in the jejunum of mice (*n* = 3). (H) The jejunum tissue was stained with H&E. Data were analyzed with Mann–Whitney *U* test (G), one-way ANOVA (C and D), or Kruskal–Wallis (B, E, and F). (B) to (D), (F), and (G) are represented as mean ± SD. (E) is represented as mean ± SEM. *P < 0.05, ***P < 0.001.

We then asked whether food allergies aggravated by melatonin could be suppressed by MT2 inhibition. Melatonin-treated food-allergic mouse was intraperitoneally injected with 4-P-PDOT, along with Abx treatment (Fig. 6A). Compared with Mel + Abx + OVA groups, 4-P-PDOT treatment relieved the allergy symptoms and reduced the allergy score and serum MCPT-1 level (Fig. 6B to E and Fig. S6A to D). 4-P-PDOT inhibited the phosphorylation of ERK and the expression of FcεRIα in the jejunum (Fig. 6F and G). Additionally, 4-P-PDOT alleviated the jejunal inflammation in melatonin-treated food-allergic mice with gut microbiota depletion (Fig. 6H). Taken together, these results indicate that MT2 inhibition alleviates food allergies in melatonin-treated mice with microbiota depletion. Moreover, melatonin also increased the expression of FcεRIα in the jejunum of mice without Abx treatment (Fig. S6E), suggesting that Mel–MT2–FceR1 signaling is still a potential therapeutic target for food allergy in normal mice.

## Discussion

Accumulating evidence shows that melatonin metabolism is altered in patients with food allergies [8–10]. Moreover, activated mast cells produce melatonin, and the expression of melatonin receptors is up-regulated in activated mast cells [33], indicating that melatonergic signaling is involved in the activation of mast cells and the pathogenesis of food allergies. Although previous studies showed that melatonin treatment relieves symptoms of allergic diseases [20,23,44], the symptoms of many allergic diseases (e.g., allergic rhinitis [21] and bronchial asthma [22]) are intense during the peak of melatonin secretion. Thus, whether and how melatonergic signaling influences mast cells and food allergies remains to be investigated. Here, our study reported that melatonin treatment relieves symptoms of food allergies but aggravates the allergic symptoms in gut microbiota-depleted mice by promoting the degranulation of mast cells. Notably, we demonstrated that melatonin enhances MT2–ERK–Rabenosyn-5 signaling to facilitate FcεRI recycling, promoting the degranulation of mast cells. These results highlight that melatonergic signaling plays an essential role in the pathological mechanism of food allergy. Moreover, our results indicated that melatonin relieves food allergy through gut microbiota, but the underlying mechanisms remain to be explored in the future.

Melatonin is mainly synthesized and secreted by the pineal gland, and blood melatonin levels exhibit rhythm, with the highest levels at night and baseline levels during the day [16]. However, the melatonin rhythm is disrupted [8] in patients with allergic disease. Moreover, a significant proportion of allergic patients suffer sleep disorders and abnormal circadian rhythmicity, which are associated with melatonin secretion disturbances [10,45]. Additionally, sleep deprivation disrupts the secretion of melatonin and increases susceptibility to food antigens [46]. These studies implicate a potential role for melatonin in food allergies. Indeed, available evidence indicates that melatonin treatment relieves symptoms of allergic diseases, such as allergic asthma [20] and atopic dermatitis [47]. Consistently, we found that supplementation of melatonin (0.2 mg/ml) in drinking water relieves symptoms of food allergies. Unexpectedly, melatonin treatment aggravated allergic reactions in mice with gut microbiota depletion. These reports indicated that melatonin suppresses food allergies by remodeling gut microbiota but supports food hypersensitivity independent of gut microbiota. However, the mechanisms of melatonin supporting food allergies under the condition of gut microbiota depletion are still unavailable.

After reexposure to food antigens, antigen-presenting cells induce a T_H_2 response that drives IgE switching in B cells and promotes the degranulation of mast cells [24]. We have revealed that melatonin treatment reduced the level of serum T_H_2 cytokines, the concentration of inflammatory mediators, and mRNA expression of chemokine receptors of mast cells in food-allergic mice. Consistent with our results, a recent study demonstrated that melatonin (10 mg/kg) intranasal treatment alleviates lung inflammation in influenza A virus (IAV) HINI-infected mice by suppressing the activation of mast cells [48]. Melatonin regulates multiple pathophysiological events through gut microbiota, and gut microbiota plays a key role in the pathogenesis of food allergy [49]. Thus, gut microbiota-depleted mice were used to confirm the direct roles of melatonin on immune response in food allergy. Interestingly, melatonin failure affects T_H_2 response and the migration of mast cells but promotes the activation of mast cells in mice with gut microbiota depletion. These results imply that melatonin promotes the activation of effector cells (e.g., mast cells) in food-allergic mice independently of gut microbiota. However, contrary results were found in another model of mast cells. A recent study found that 10 μM melatonin pretreatment inhibited the release of proinflammatory mediators by HIN1 virus-infected mouse mast cell line P815 [48]. These seemingly contradictory results may be due to differences in cell source and pathological model. The classic trigger for mast cell degranulation in food allergies is through IgE bound to high-affinity IgE receptors, FcεRI [36], but HIN1-induced mast cell degranulation is independent of IgE–FcεRI signaling [48], which further implies that melatonin targets IgE–FcεRI signaling on mast cell in food-allergic mice. Meanwhile, mast cells are the main effector cells of IgE-mediated allergic reactions to foods. Thus, whether and how melatonin affects mast cells remains to be further elucidated.

In this study, we found that melatonin promotes the activation of mast cells through up-regulated expression of membrane FcεRI. Recent studies showed that the endolysosomal system is involved in the regulation of the abundance of cell surface proteins through the degradative pathway and endocytic recycling pathway [50]. Notably, about 70 to 80% of endocytosed material is recycled to the plasma membrane via recycling endosomes [39]. Here, we showed that melatonin up-regulates the expression of membrane FcεRI through endocytic recycling. FERARI cooperates with SNX6 to regulate sorting endosomes, thereby coordinating the vectorial flow of cargo in endocytic recycling through sorting endosomes [39,42]. FERARI contains multiples proteins, including Rabenosyn-5, vacuolar protein sorting 45 homolog (VPS45), vps33b interacting protein, apical–basolateral polarity regulator, SPE-39 homolog (VIPAS39), ankyrin 1 (ANK1), and eps15 homology domain protein 1 (EHD1) [42]. It should be mentioned that Rabenosyn-5 mediates the interaction between FERARI and SNX6 [42]. Correspondingly, we observe that melatonin facilitates FcεRI recycling by elevating the mRNA expression of Rabenosyn-5. However, it is still unclear how melatonin affects the mRNA expression of Rabenosyn-5. Recent evidence indicates that the phosphorylation of ERK supports the function of endosomal recycling [43], and melatonin leads to ERK activation via MT2 [2]. Currently, our results demonstrated that inhibition of MT2 signaling blocks the promotion effects of melatonin on FcεRI recycling and mast cell degranulation in vivo and in vitro. Therefore, it would be interesting to further explore how MT2/ERK mediates the expression of Rabenosyn-5 in mast cells.

In conclusion, we have elucidated that melatonin supports food allergies in mice with gut microbiota depletion. Mechanistically, melatonin facilitates FcεRI recycling through the MT2–Rabenosyn-5 signaling, thereby promoting the degranulation of mast cells, and this process may be mediated by ERK signaling. Hence, manipulating the melatonergic system may be an effective strategy for preventing and treating food allergies in the future.

## Methods

### Mice

All animal experiments were approved by the Laboratory Animal Ethical Commission of South China Agricultural University. Five-week-old female BALB/c mice were obtained from SiPeiFu Biotechnology (China). All mice were kept in standard housing with a 12-h light–dark cycle at 22 °C and kept on an OVA-free diet and water ad libitum.

### Cell lines

RBL-2H3 were cultured in complete Dulbecco’s modified Eagle’s medium (DMEM). RBL-2H3 cells were sensitized overnight at 37 °C with anti-DNP IgE (Sigma-Aldrich, 250 ng/ml) in complete DMEM. The following day, cells were washed with phosphate-buffered saline (PBS) and stimulated with DNP-HSA (Sigma-Aldrich, 500 ng/ml) for 30 min at 37 °C.

### Food allergy model

For sensitization, mice were intraperitoneally injected with 50 μg of OVA (Merck, USA) together with 1 mg of Imject alum adjuvant (Thermo Fisher Scientific, USA) in 200 μl of sterile PBS at days 1 and 14. On the fourth week, mice were challenged intragastrically with 50 mg of OVA in 200 μl of PBS for 6 times at 2-day intervals (days 28 to 38). As a vehicle control, mice were intraperitoneally injected with 1 mg of Imject alum adjuvant in 200 μl of PBS at days 1 and 14. On the fourth week, mice received 200 μl of PBS by intragastric gavage 6 times at 2-day intervals (days 28 to 38). Mice were fasted for 4 h before oral challenge with OVA. The evaluation of food allergy score was based on body weight gain and diarrhea occurrence. The body weight of the mice was recorded before the first and sixth OVA intragastric challenges in a food allergy model to calculate the body weight gain. The diarrhea occurrence of mice was assessed by visually monitoring mice for up to 1 h after oral challenge. A detailed clinical score was assessed in Fig. S1 and Table S1. Diarrhea score = *A*; number of diarrhea = *B*; body weight gain score = *C*; allergy score = *A* + *B* + *C* − (*A*_Cont_ + *B*_Cont_ + *C*_Cont_)/*n*.

### Microbiota depletion

Mice were treated with antibiotics via drinking water (0.5 g/l vancomycin, 1 g/l gentamicin, 1 g/l streptomycin, and 1 g/l ampicillin) 3 times for 5 days each time to deplete the gut microbiota.

### Drug administration

In some experiments, mice were treated with 0.2 mg/ml melatonin (Sangon Biotech) in drinking water. The melatonin water was kept in foil-coated bottles to prevent melatonin degradation. In some experiments, 4-P-PDOT (MedChemExpress) was intraperitoneally injected at doses of 0.5 mg/kg before the OVA intragastric challenge.

### FcεRIα or Rabenosyn-5 knockdown in RBL-2H3 cells

RBL-2H3 cells were cultured at a density of 4 × 10^5^ in a 6-well plate and transfected with FcεRIα or Rabenosyn-5 small interfering at 200 ng by using the Lipofectamine 3000 transfection reagent.

### ELISA for total and OVA-specific IgE and murine MCPT-1

The concentration of total and OVA-specific IgE and MCPT-1 in serum was measured by using enzyme-linked immunosorbent assay (ELISA) kits (mouse IgE ELISA kit, Abcam; mouse serum anti-OVA IgE antibody assay kit, Chondrex; MCPT-1 mouse uncoated ELISA kit, Thermo Fisher Scientific) according to the manufacturer’s protocol.

### Tissue histological analysis

Mouse jejunum was fixed in 4% paraformaldehyde overnight. Then, jejunal tissues were embedded in paraffin and sectioned at 4 μm. Finally, the jejunal sections were stained with hematoxylin and eosin (H&E) staining.

### Histamine and melatonin measurements

The level of histamine and melatonin in serum and supernatants from stimulated mast cell cultures was quantified with targeted metabolomics as in our previous study [51].

### Enzyme activity of ASMT measurements

The enzyme activity of ASMT was measured as in a previous study [52]. Briefly, cells were washed with precooled PBS and then repeatedly frozen-thawed 5 times to lyse the cells. For determination of ASMT activity, cell extracts in 100 μl of PBS were assayed in100 μl of 100 mM PBS (pH 7.8), 1 mM N-acetylserotonin, and 1 mM S-adenosyl-L-methionine. After incubation at 37 °C for 1 h, the reaction was stopped with 50 μl of methanol. Finally, the melatonin product in the reaction system was detected.

### Western blot

Jejunum tissue or RBL-2H3 cells were lysed with ice-cold radioimmunoprecipitation assay (RIPA) buffer (plus 2% protease and 2% phosphatase inhibitor) for 10 min. Then, Western blot was performed as in our previous study [51]. Antibodies against ERK (11257-1-AP), p-ERK (28733-1-AP), β-actin (66009-1-lg), and glyceraldehyde-3-phosphate dehydrogenase (GAPDH) (60004-1-lg) were purchased from Proteintech (Rosemont, USA).

### Confocal immunofluorescence staining

Paraffin-embedded slides or cells were examined with immunofluorescence staining for FcεRIα (Thermo Fisher Scientific, A5-115221), ATP1A1 (Proteintech, 55187-1-AP), giantin (Abcam, ab37266), Rab5 (Cell Signaling, 3547), Rab7 (Cell Signaling, 9367), Rab4 (Abcam, ab109009), and Rab11 (Cell Signaling, 5589). Images were observed under a confocal fluorescence microscope (Zeiss) and analyzed using ImageJ.

### Real-time quantitative PCR

RNA Purification Kit (EZBioscience, USA) was used to extract RNA from Jejunum tissues or RBL-2H3 cells. Color Reverse Transcription Kit (EZBioscience) was used for reverse transcription. The 2× Color SYBR Green qPCR Master Mix (EZBioscience) was used to perform the quantitative polymerase chain reaction (PCR). Data were analyzed by using the 2^−∆∆*C*t^ method with β-actin. The primers used in the study are listed in Table S2.

### Bacterial DNA isolation and 16*S* rDNA gene sequence analysis

DNA extracted from murine feces was performed using the hexadecyl trimethyl ammonium bromide (CTAB)/sodium dodecyl sulfate (SDS) method. The 16*S* rRNA genes of V3–V4 regions were amplified using specific primers with the barcode. TruSeq DNA PCR-Free Sample Preparation Kit (Illumina) was used to generate the sequencing libraries. The Qubit@ 2.0 Fluorometer (Thermo Fisher Scientific) and Agilent Bioanalyzer 2100 system were used to assess the library quality. Finally, amplicon library was sequenced on an Illumina NovaSeq platform.

### Statistical analysis

All data were analyzed and performed with Prism 8.0 software (GraphPad), and results are represented as means ± SEM or SD. Kaplan–Meier analysis was used to evaluate the diarrhea occurrence of mice. Unpaired *t* test analysis was used to assess data between 2 groups in Gaussian distribution and had equal variance. Nonparametric test (Mann–Whitney *U* test) was used to assess data between 2 groups not normally distributed. One-way analysis of variance (ANOVA) followed by Dunnett multiple comparisons was used to assess data from more than 2 groups in Gaussian distribution and had equal variance. Kruskal–Wallis followed by Dunn’s multiple comparisons was used to assess data from more than 2 groups not normally distributed. PCoA was performed with the R software. Unweighted pair-group method with arithmetic means (UPGMA) clustering was performed with the QIIME software. *P* < 0.05 indicates statistical significance.

## Data Availability

The data reported are present in the paper or the Supplementary Materials. The 16*S* data have been deposited in the Genome Sequence Archive database in China National Center for Bioinformation (CRA014665 and CRA014670).
