# Supplementary material for: Melatonergic Signaling Sustains Food Allergy Through FcεRI Recycling
Source: Research (Wash D C). 2024 Jul 22;7:0418. doi: 10.34133/research.0418 (PMC11260513; doi:10.34133/research.0418)
Supplement: Supplementary 1 — Tables S1 and S2 Figs. S1 to S6 [file research.0418.f1.zip › Mel mast cell Supplementary Materia-2024.5.28l.docx]

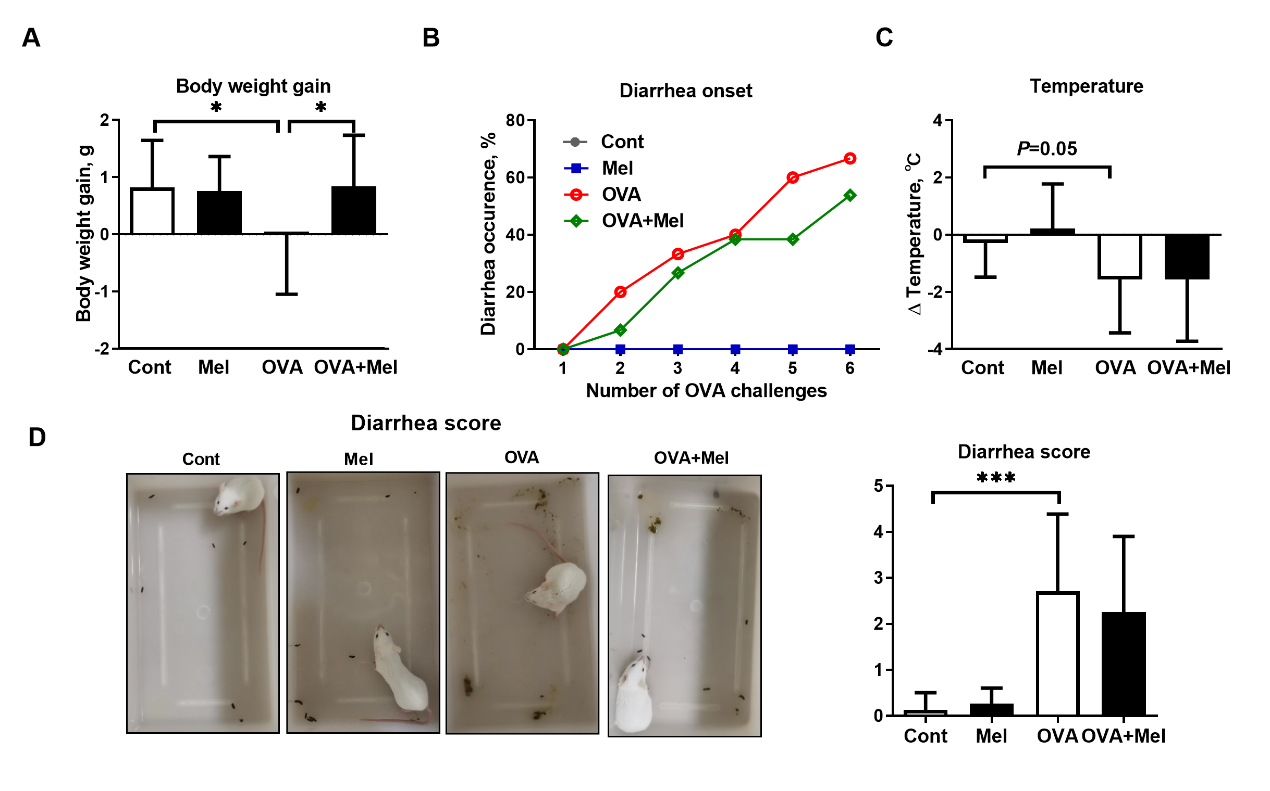


**Figure. S1. Melatonin relieves clinical symptoms of food allergy.** (**A**) Body weight gain during allergy of mice (n=13-15). (**B**) The occurrence of diarrhea was monitored for 60 minutes after each *i.g.* administration (n=13-15).(**C**) The rectal temperature was measured 30 minutes after the last *i.g.* exposure on day 38 (n=13-15). (**D**) Diarrhea score of mice (n=13-15). Data were analyzed with Kaplan-Meier analysis (B) or Kruskal-wallis (A, C and D). Data represented as mean ± SD. **P* < 0.05, ****P* < 0.001.


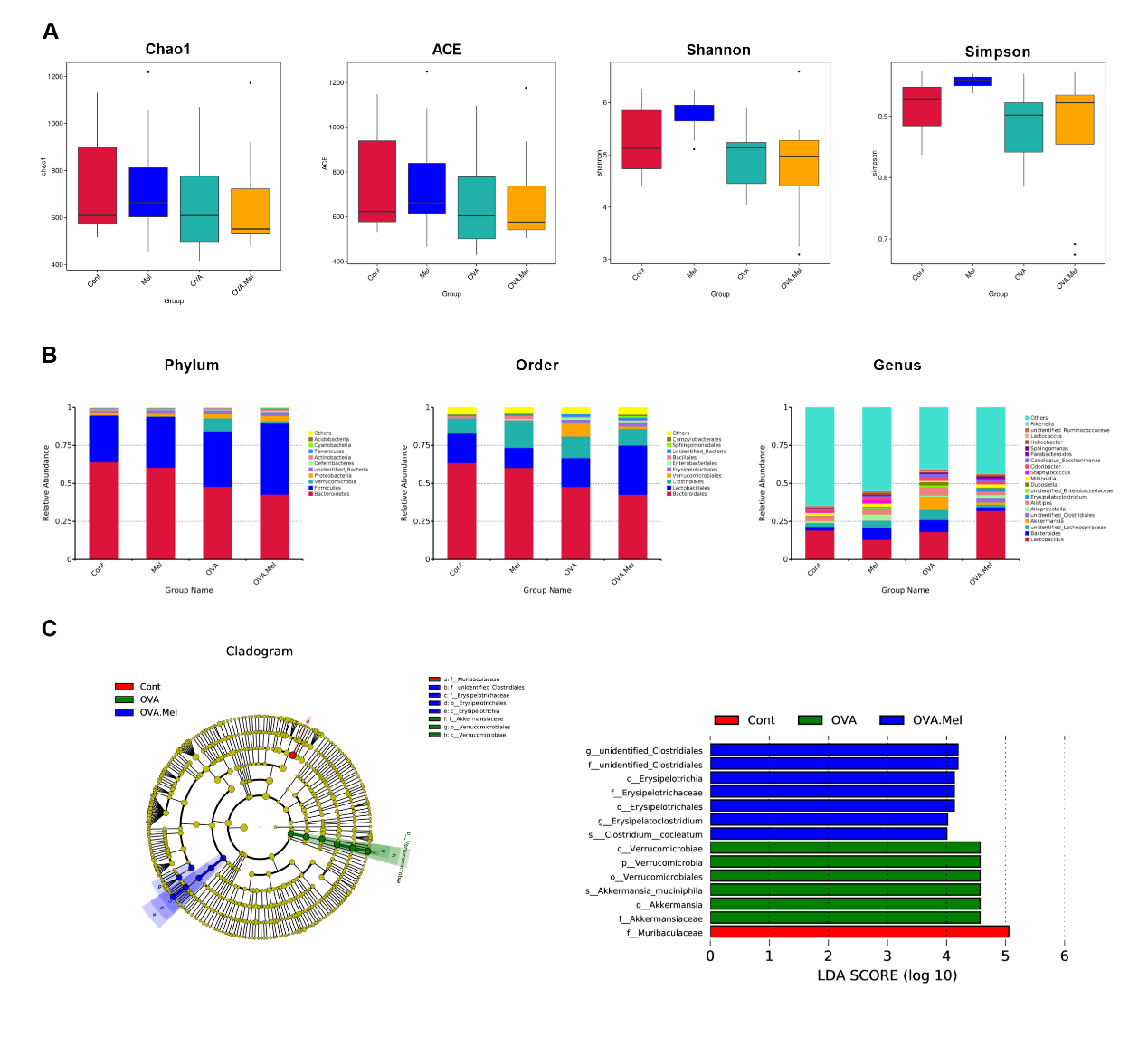


**Figure. S2. Melatonin modulates gut microbiota composition of OVA-induced food allergy.** (**A**) Chao1, ACE, Shannon and Simpson index in α-diversity analysis of the mice gut microbiota (n=9-11). (**B**) Microbiota composition at the phylum, order, and genus level of mice (n=9-11). (**C**) LEfSe analysis of Illumina MiSeq sequencing data obtained from each group (n=9-11).


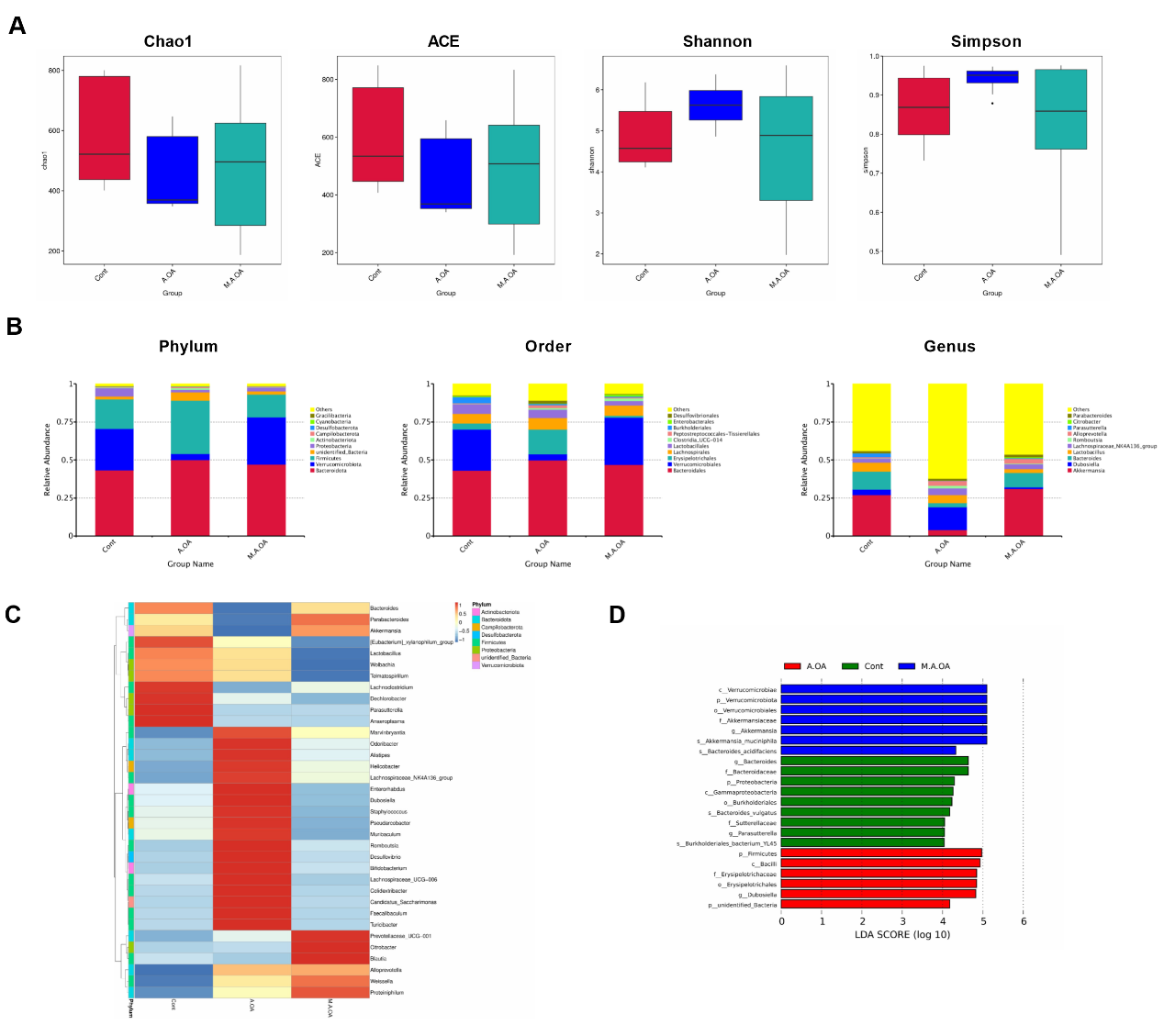


**Figure. S3. Gut microbiota composition of food allergy mice treated with antibiotics.** (**A**) Chao1, ACE, Shannon and Simpson index in α-diversity analysis of the mice gut microbiota (n=10). (**B**) Microbiota composition at the phylum, order, and genus level of mice (n=10). (**C**) The heatmap showed the gut microbiota composition at the phylum level of mice (n=10)**.** (**D**) LDA analysis of Illumina MiSeq sequencing data obtained from each group (n=10).


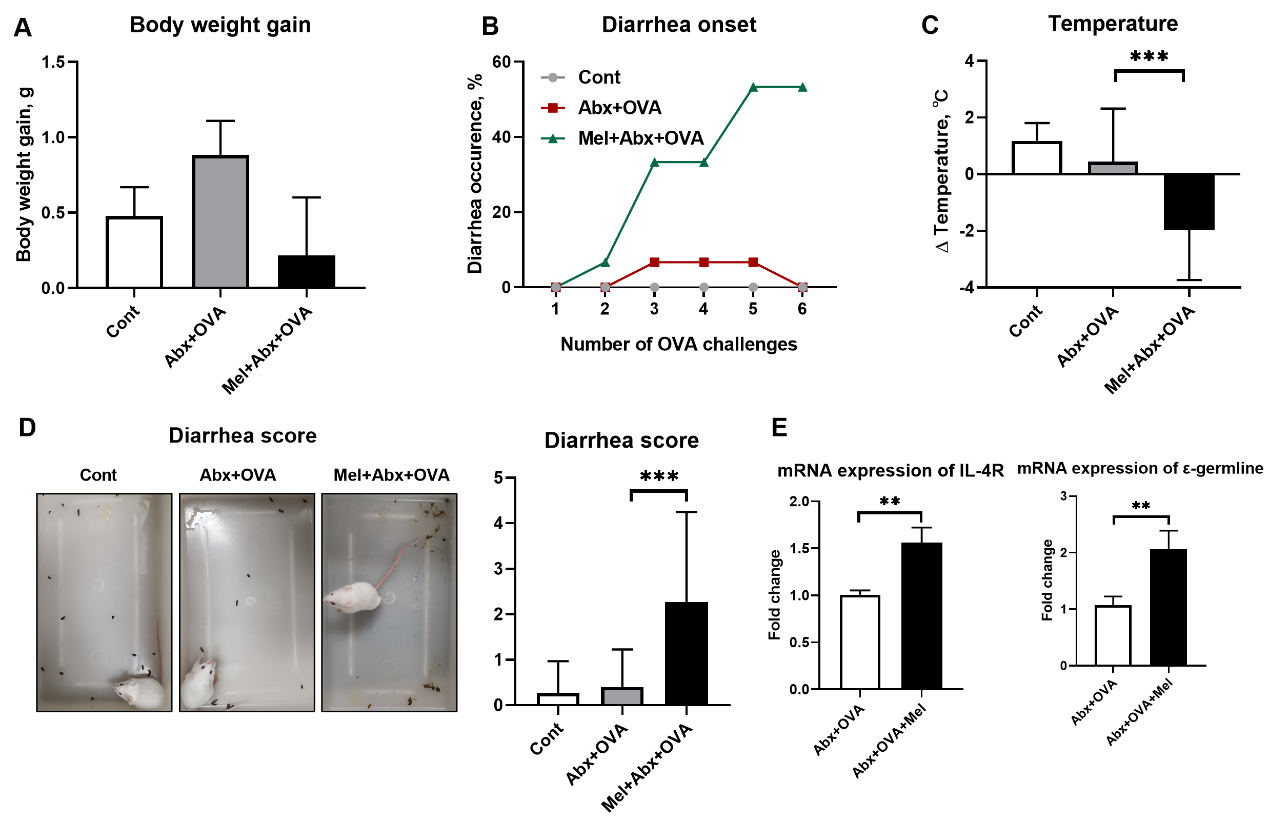


**Figure. S4. Melatonin exacerbates the clinical symptoms of food allergy in mice with gut microbiota depletion.** (**A**) Body weight gain during allergy of mice (n=14-15). (**B**) The occurrence of diarrhea was monitored for 60 minutes after each *i.g.* administration (n=14-15). (**C**) The rectal temperature was measured 30 minutes after the last *i.g.* exposure on day 45 (n=14-15). (**D**) Diarrhea score of mice (n=14-15). (**E**) Relative mRNA expression of IL-4R and ε-germline (n=4). Data were analyzed with Kaplan-Meier analysis (B), Kruskal-wallis (A, C and D) or Mann-Whitney *U* test (E). Data represented as mean ± SD. ****P* < 0.001.


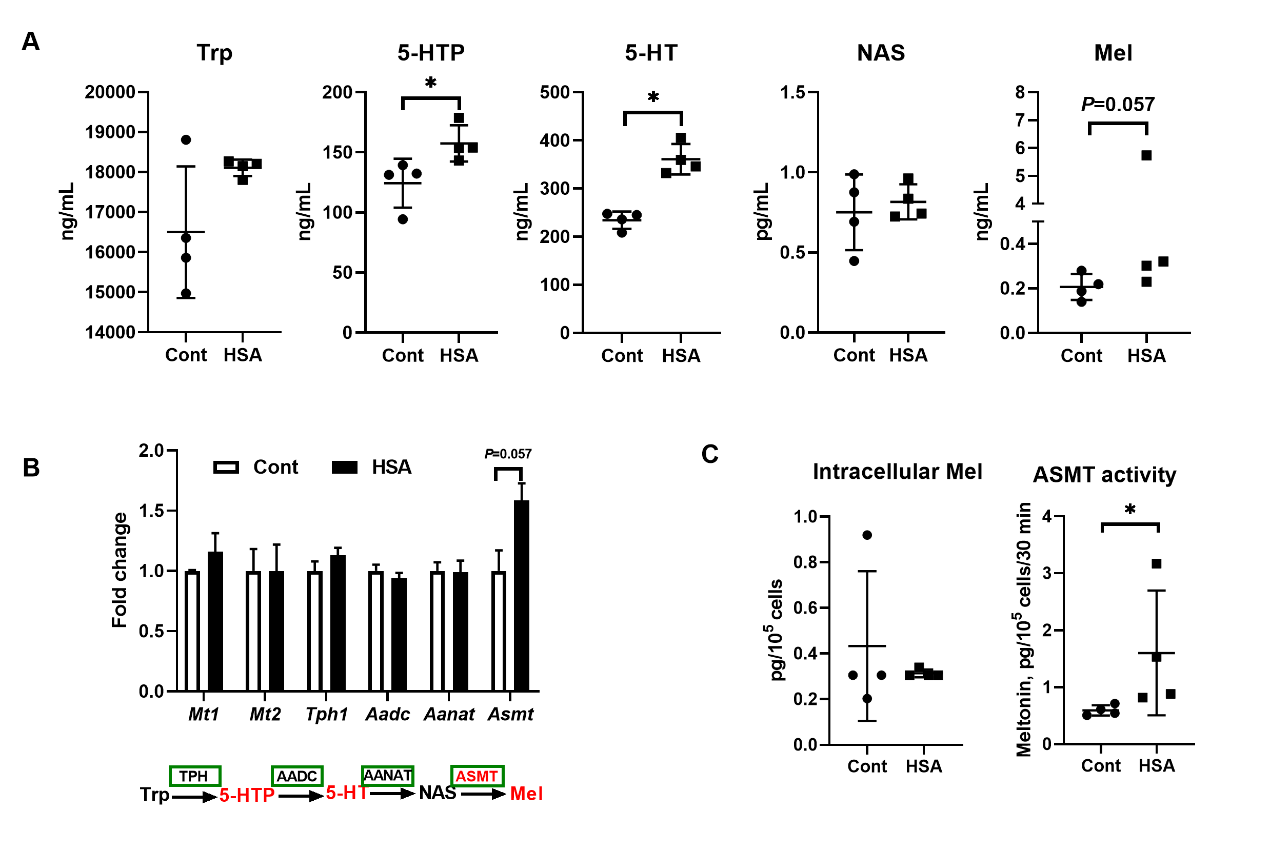


**Figure. S5. Activated mast cells showed altered melatonin metabolism.** (**A**) The concentration of supernatant metabolites for melatonin synthesis in mast cells stimulated with or without DNP-HSA (n=4). (**B**) Relative mRNA expression of enzymes for melatonin synthesis in mast cells stimulated with or without DNP-HSA (n=4). (**C**) Enzymatic activity of ASMT in mast cells stimulated with or without DNP-HSA (n=4). Data were analyzed with Mann-Whitney *U* test. A and C represented as mean ± SD. B represented as mean ± SEM. **P* < 0.05.


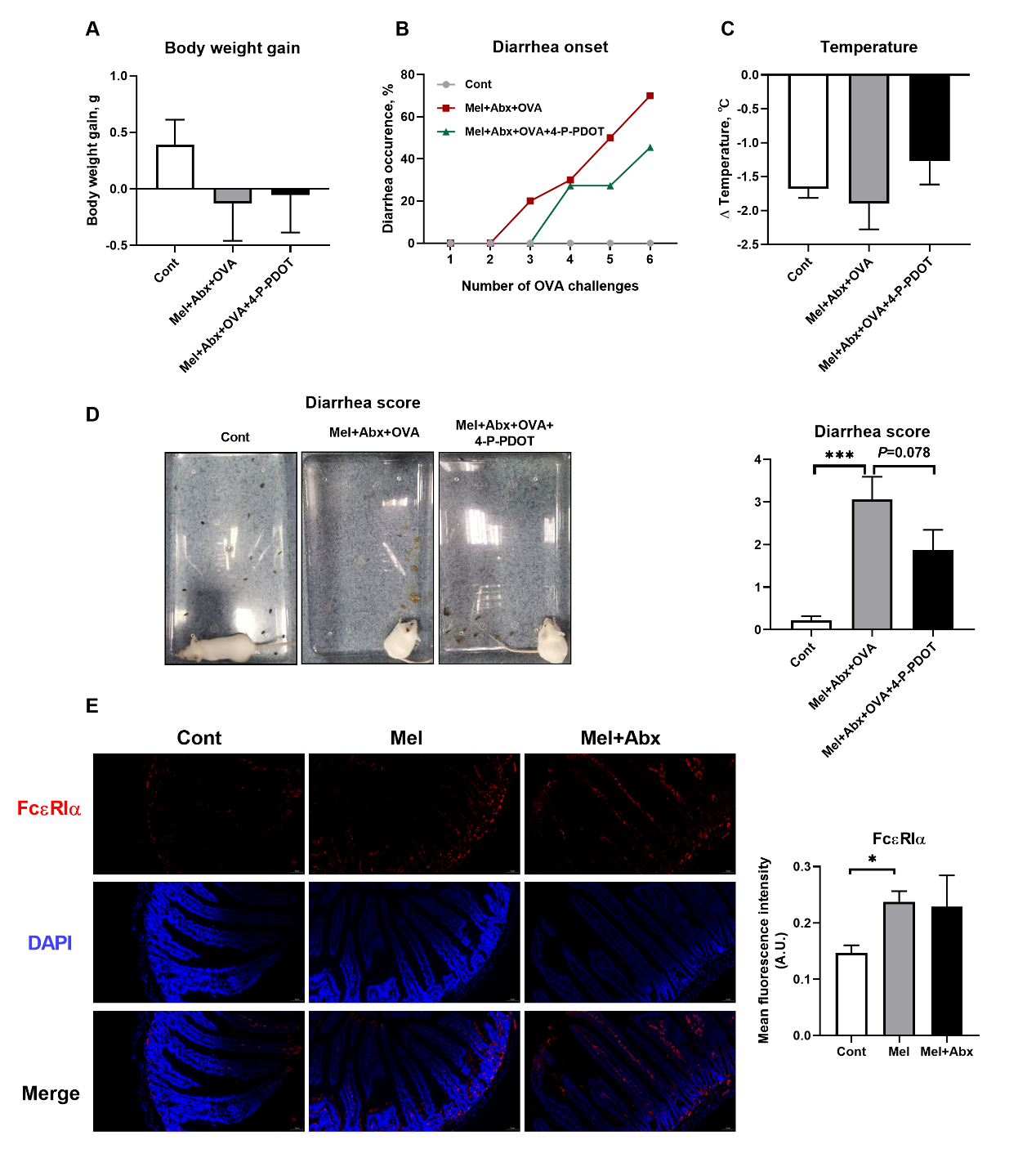


**Figure. S6. MT2 inhibition attenuates the clinical symptoms of melatonin-induced food allergies in mice with gut microbiota depletion.** (**A**) Body weight gain during allergy of mice (n=10-12). (**B**) The occurrence of diarrhea was monitored for 60 minutes after each *i.g.* administration (n=10-12). (**C**) The rectal temperature was measured 30 minutes after the last *i.g.* exposure on day 45 (n=10-12). (**D**) Diarrhea score of mice (n=10-12). (**E**) Confocal microscopy of FcεRIα (green) in the jejunum of mice (n=3). Data were analyzed with Kaplan-Meier analysis (B) or Kruskal-wallis (A, C, D and E). Data represented as mean ± SD. ****P* < 0.001.

**Table S1. The detailed description of allergy score.**

| **Items** | **Diarrhea score** | **Number of diarrheas** | **Body weight gain score** |
| --- | --- | --- | --- |
|  | Normal =0 | 0 | ＞0.6 g =0 |
|  |  | 1 | 0.45≤A≤0.60 g =1 |
|  | Soft =2 | 2 | 0.30≤A≤0.45 g =2 |
|  |  | 3 | 0.15≤A≤0.30 g =3 |
|  | Liquid =4 | 4 | <0.15 g =4 |

**Table S2. Primers used for mRNA expression analysis by RT-PCR.**

| **Gene names** | **Forward Primer (5’ to 3’)** | **Reverse Primer (5’ to 3’)** |
| --- | --- | --- |
| Mouse *Il4* | AACGAGGTCACAGGAGAAGG | TGGAAGCCCTACAGACAAGC |
| Mouse *Il5* | CCCTCATCCTCTTCGTTGC | ATCCTCCTGCGTCCATCTG |
| Mouse *Il13* | AGACCAGACTCCCCTGTGCA | TGGGTCCTGTAGATGGCATTG |
| Mouse *Scf* | AGCTTGACTACTCTTCTGGACA | TGGCCTCTTCGGAGATTCTTTT |
| Mouse *Cxcr2* | GGTCGTACTGCGTATCCTGCCTCAG | TAGCCATGATCTTGAGAAGTCCATG |
| Mouse *Cxcl1* | CTGGGATTCACCTCAAGAACATC | CAGGGTCAAGGCAAGCCTC |
| Mouse *Cxcl2* | AGTGAACTGCGCTGTCAATGC | AGGCAAACTTTTTGACCGCC |
| Rat *Fcer1a* | GGCTGCTGCTCCAATCTTC | GCAATGTCGTCCTTGTAGTAGA |
| Rat *Ms4a2* | TGCTCCACACTCCAGACTTC | GCTGCCTCTCACCAGATACA |
| Rat *Fcer1g* | GGTGATCTTGTTCTTGCTCCTT | TCACGGCTGGCTATGTCTG |
| Rat *SNX1* | GGTGGAGGAAGATGGCGTC | GACTGGGGCTTACTAGCTGC |
| Rat *SNX6* | AAGTGGACATTTCGGACGCT | TGGTGCTGGTGGGATAATGT |
| Rat *Ehd1* | AAGCTTAACGCCTTCGGCAA | CCGCAAAGTCATAACCTCGG |
| Rat *Vps45* | CACACTCAGAGACAGGCCTCA | CAGAAGCCAGGACTTCCTCTA |
| Rat *Rab11FIP5* | CCTCTGCTCTGCTGCTGGAAG | CCATCTTGACCGCTAACAACCTTG |
| Rat *Rabenosyn-5* | TCTGTCCTGACTTGGTTGCTTCC | ACTGGGTTTAGACTCTGCCTTCAC |
| Rat *Vipas39* | GCTCCAAGTTCAAGGCGTTCAC | AGGTCATCGTCATCGTCGTCATC |
| Rat *Ank1* | AACGAGGACCAAGGATGAACTGAC | CCTGAGCCGCCATGTGGATG |
| Rat *Ank2* | ACGGATGTGGATGCTGACTCTTG | GTGGTGCCCTGTGACTGTCTG |
| Rat *Ank3* | ATGAAAGCCAGTAGCGAGGAAGAG | TCGTGGTTGTGGTGATGTGAGTC |
