# Supplementary figures and images for: Melatonergic Signaling Sustains Food Allergy Through FcεRI Recycling
Source: Research (Wash D C). 2024 Jul 22;7:0418. doi: 10.34133/research.0418 (PMC11260513; doi:10.34133/research.0418)

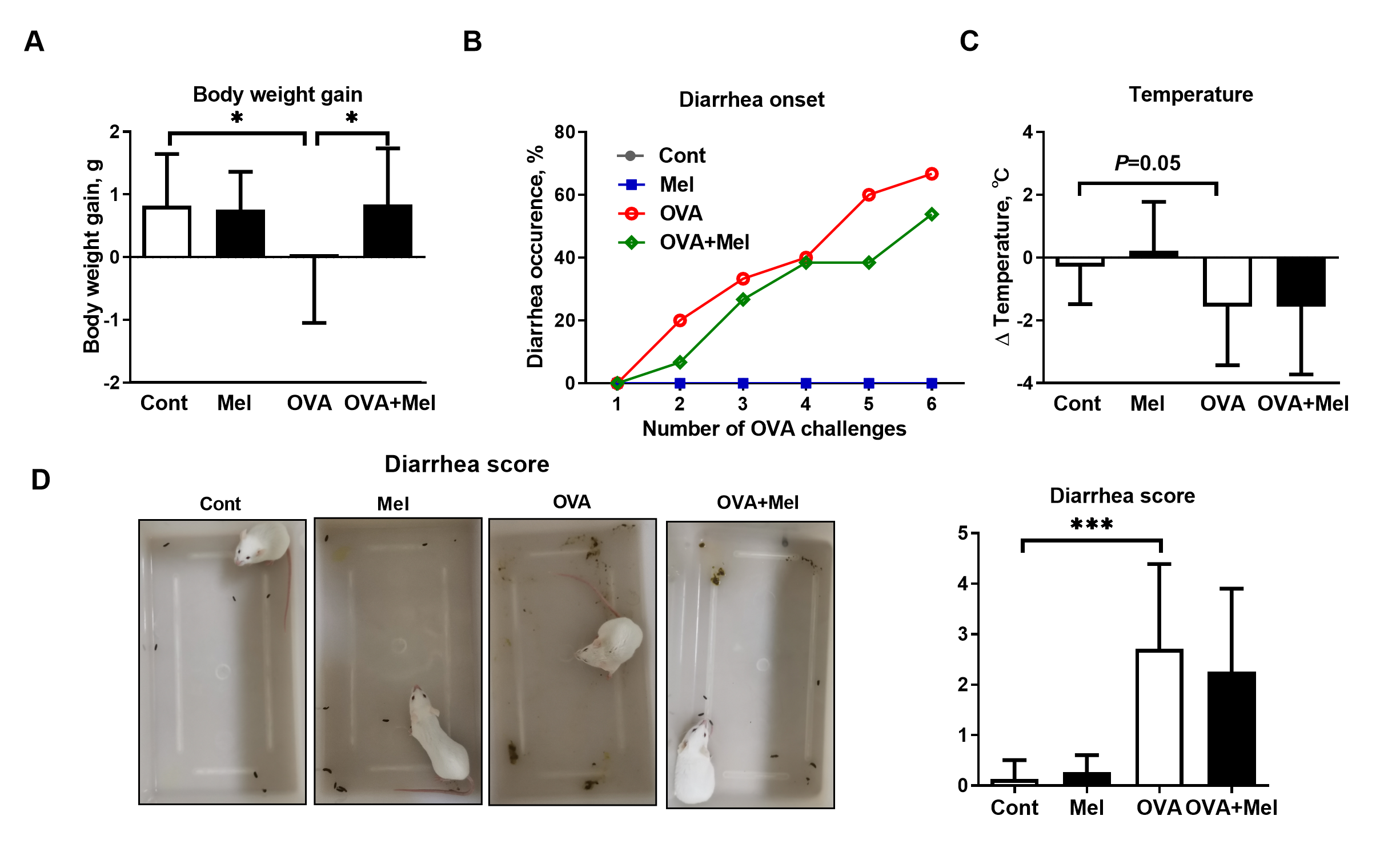

Supplement: Supplementary 1 — Tables S1 and S2 Figs. S1 to S6 [file research.0418.f1.zip › Figure S1.tif]

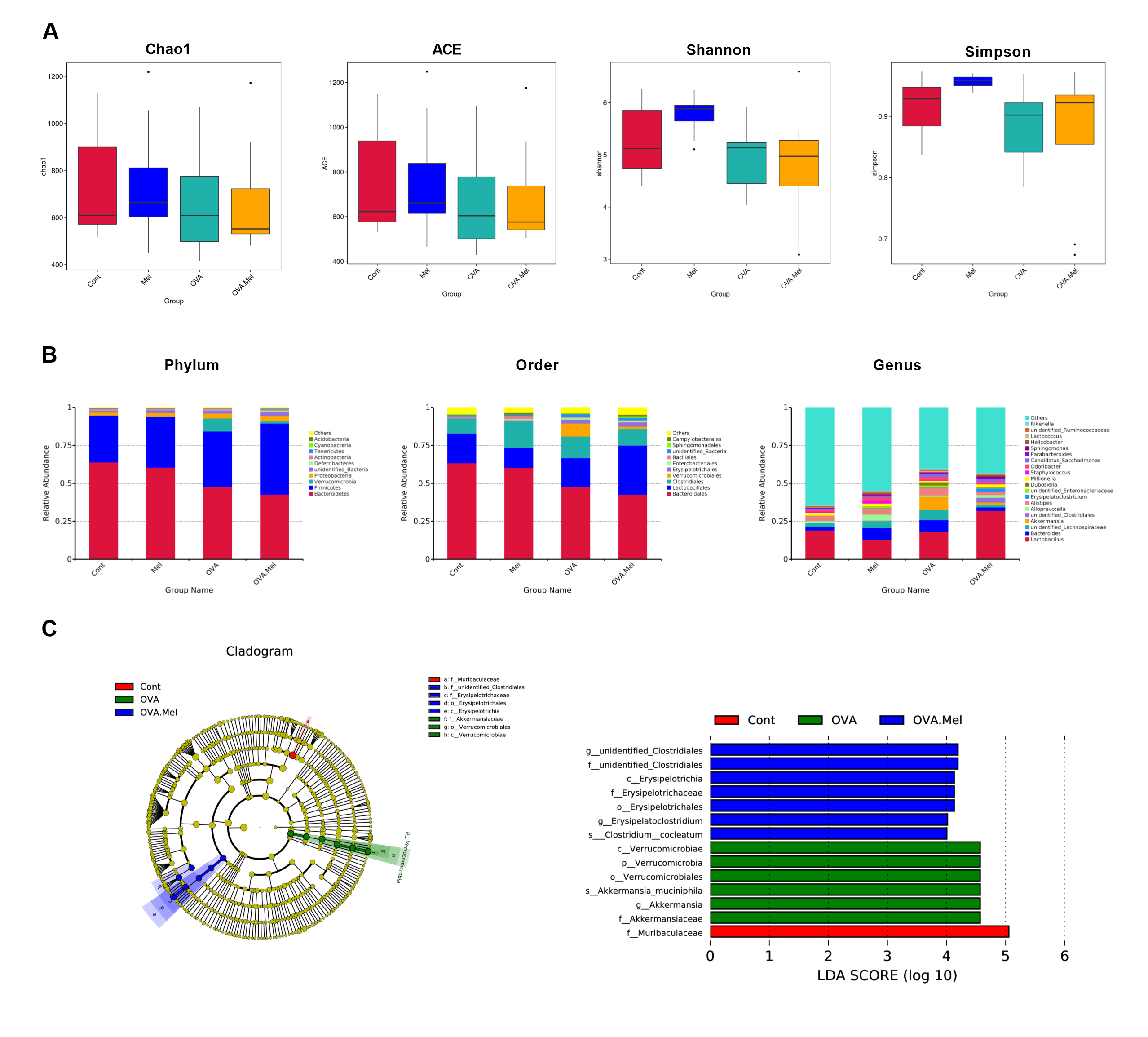

Supplement: Supplementary 1 — Tables S1 and S2 Figs. S1 to S6 [file research.0418.f1.zip › Figure S2.tif]

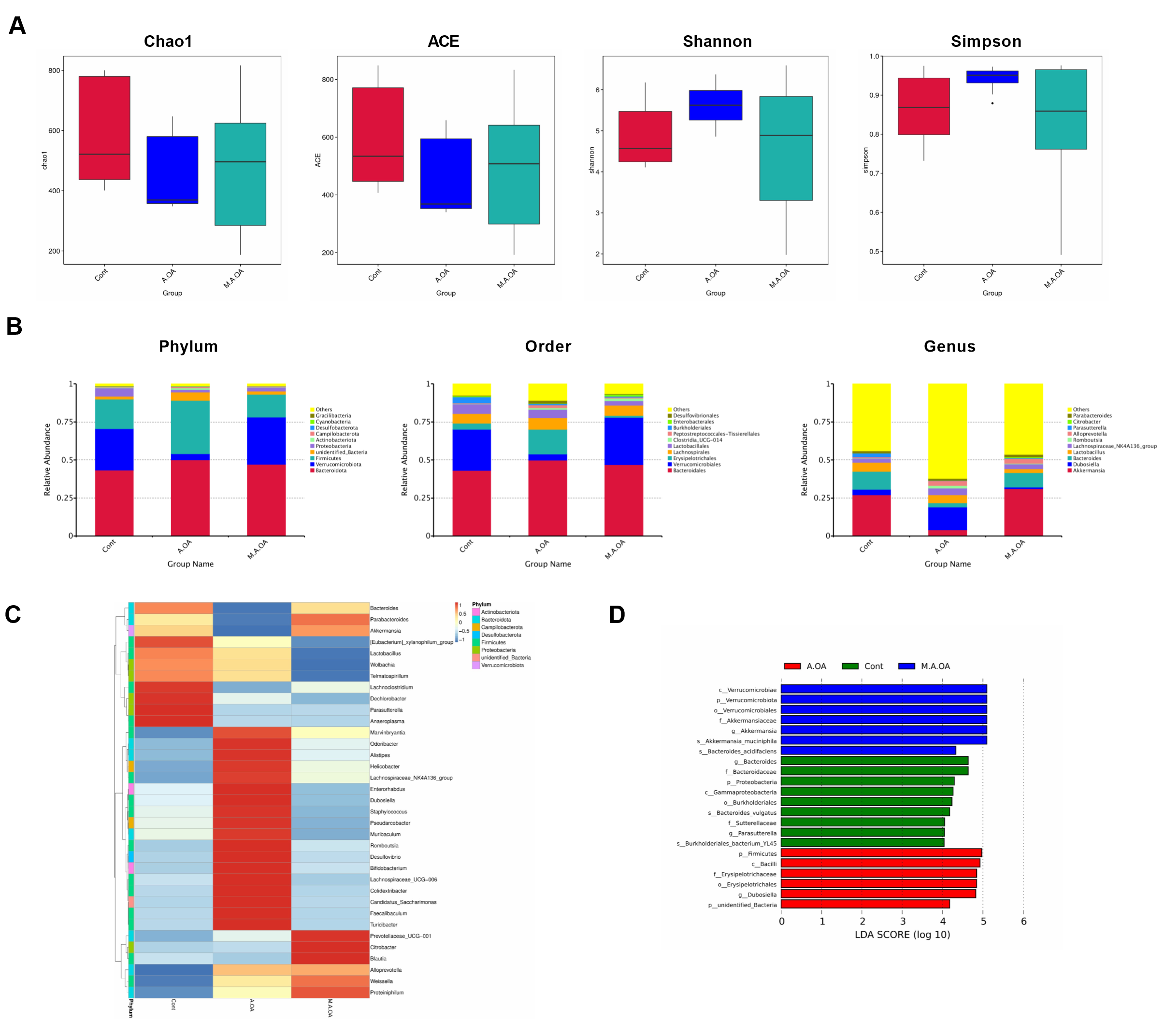

Supplement: Supplementary 1 — Tables S1 and S2 Figs. S1 to S6 [file research.0418.f1.zip › Figure S3.tif]

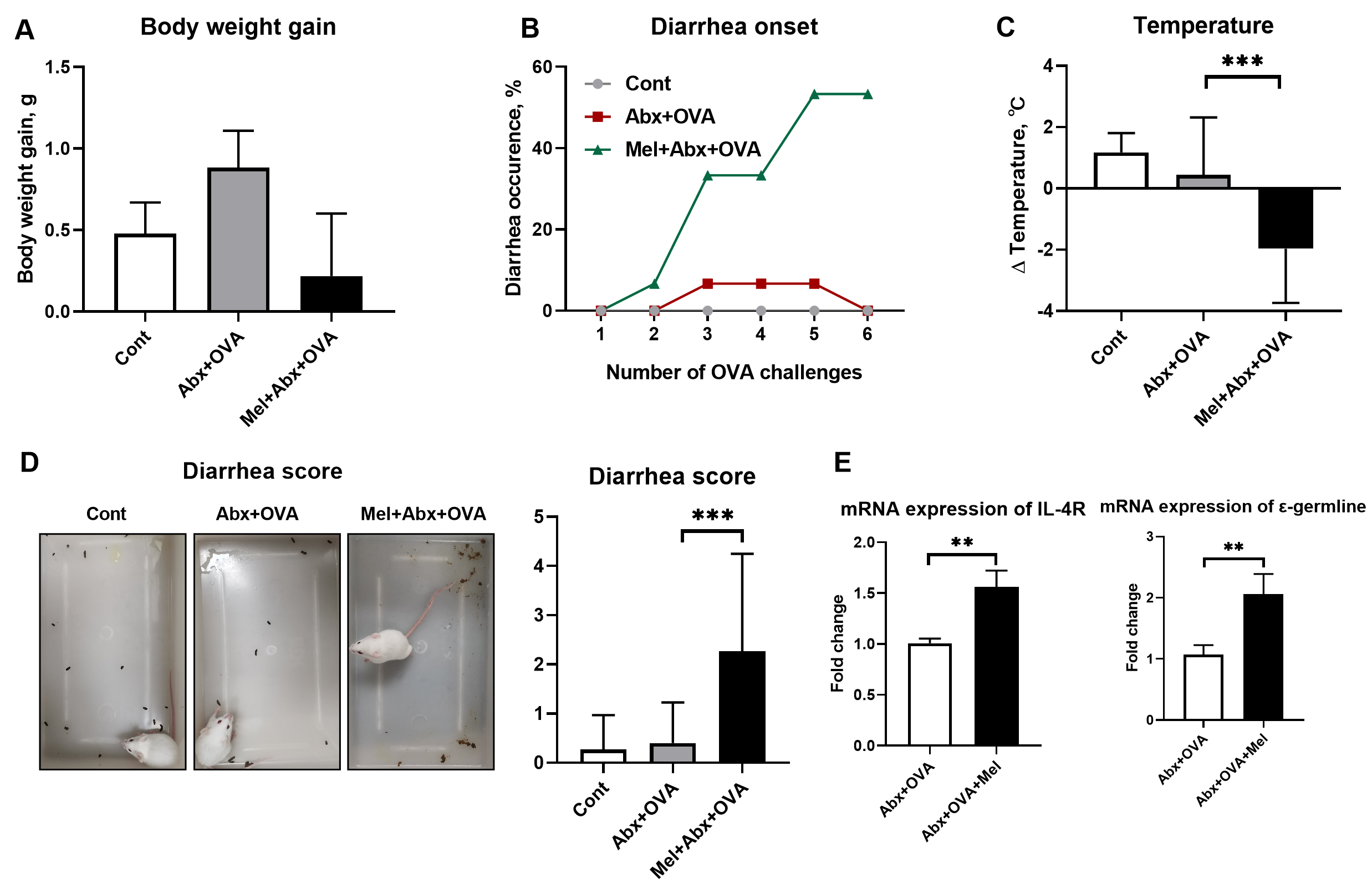

Supplement: Supplementary 1 — Tables S1 and S2 Figs. S1 to S6 [file research.0418.f1.zip › Figure S4.tif]

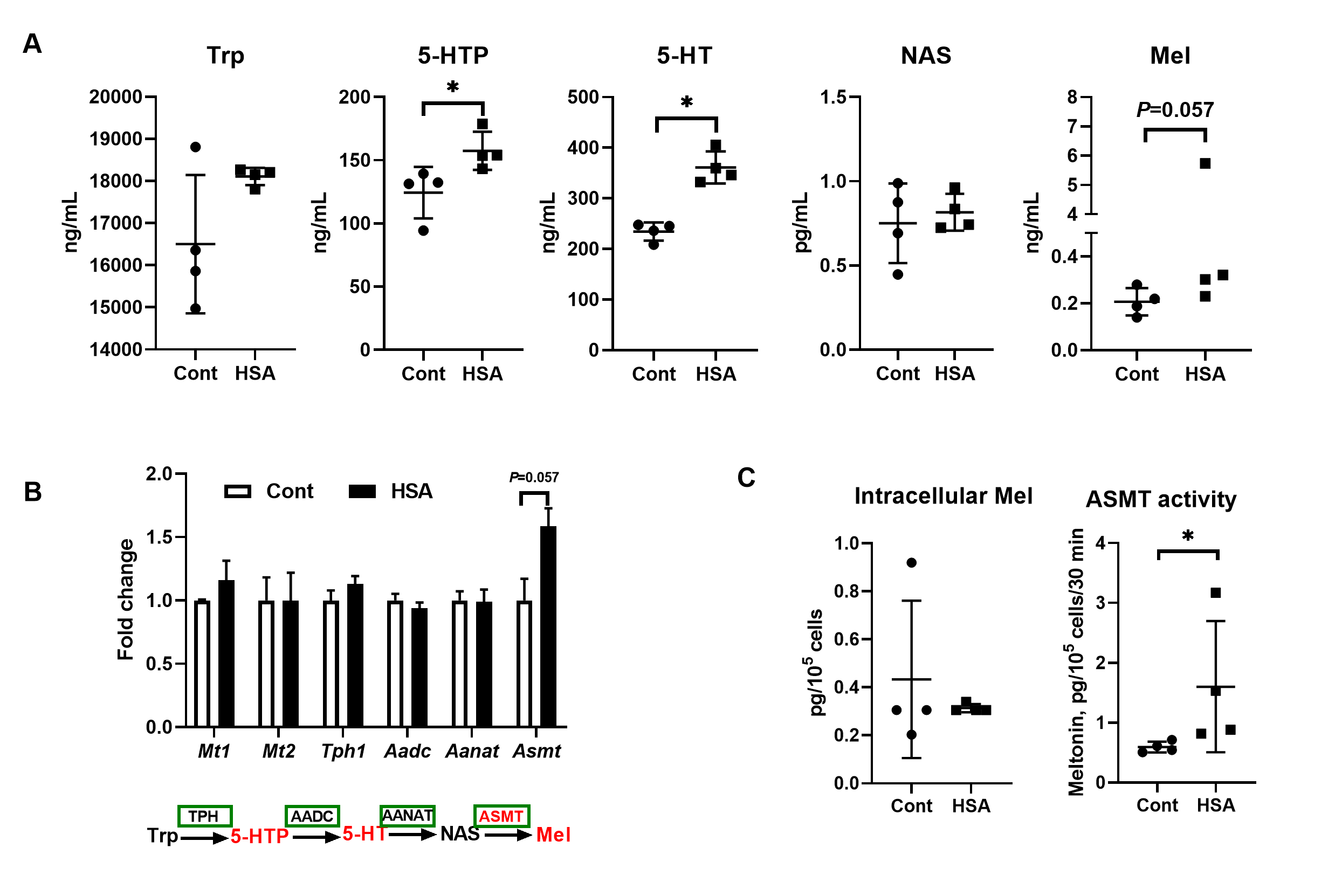

Supplement: Supplementary 1 — Tables S1 and S2 Figs. S1 to S6 [file research.0418.f1.zip › Figure S5.tif]
